# Amphotericin B® treatment causes QT prolongation in lung transplant-patients

**DOI:** 10.1186/2197-425X-3-S1-A213

**Published:** 2015-10-01

**Authors:** B-A Tudor, A Slama, GA Roth, C-G Krenn

**Affiliations:** Medical University of Vienna, Department of Anesthesiology, General Intensive Care and Pain Medicine, Vienna, Austria; Medical University of Vienna, Department of Thoracic Surgery, Vienna, Austria

## Intr

One-fifth of patients admitted to Intensive Care Units suffer from significant cardiac arrhythmias, contributing to a major source of morbidity [1]. Since arrhythmias rarely becomes obvious during admission, a close monitoring is mandatory to ensure early therapeutic intervention if necessary. However, the effects of disease pattern and intensive care measures (e.g. sepsis, medication) on heart rate and variability are poorly understood. Electrical instability could be detected during repolarization shortly before cardiac arrest with a high resolution ECG, not recordable with a conventional ECG, even in patients without prior heart pathologies [2].

## Objectives

We analysed changes of beat-to-beat cardiac activity during intensive care stay and therapeutic intervention (e.g. antimycotic therapy) of double lung transplanted recipients with a high resolution electrocardiogram. Obtained results may offer new insights in the development of alterations in cardiac electrical activity of critical ill patients.

## Methods

At 1000 Hz sampling rate the cardiac electric activity of 45 double lung transplanted recipients on ICU were analysed during their stay and inhalationtherapy with 50 mg of Amphotericin B®. The electrodes were fixed on the prepared skin for recording the leads I, II, III and reconstruct pursuant to Einthoven´s equation aVR, aVL, aVF, obtaining continuous ten-minute recordings (Lab System^Th^ Pro - Bard electrophysiology U.S.A.).

## Results

Results obtained from 45 Amphotericin B® treatments show irrespective of the prior QT range an increasing of the QT-interval additionally up to 21 ms (p < 0,05) from the beginning of the infusion. This variation was observed for two minutes of therapy and returns with a slow to their pre-value. Concerning QT prolongation the beat-to-beat duration increases too. Other ECG data remained unchanged during the time of treatment.

## Conclusions

Haemodynamic alterations could be detected after the onset of antimycotic treatment with Amphotericin® lung transplantation recipients. With regard to comorbidities of lung transplanted patients, it seems reasonable that changes in cardiac electric activity based on heart lung interactions in humans during their ICU stay and their origin might be observed even earlier. In contrast even minor circadian beat to beat variations might precede the occurrence of potentially life threatening arrhythmias.
